# “Living People Who Breathe and Feel and Suffer and Love”

**DOI:** 10.3201/eid2910.AC2910

**Published:** 2023-10

**Authors:** Byron Breedlove

**Affiliations:** Centers for Disease Control and Prevention, Atlanta, Georgia, USA

**Keywords:** syphilis, congenital syphilis, Edvard Munch, Inheritance, sexually transmitted infections, penicillin, public health, art and science, about the cover, birth defects, Treponema pallidum, bacteria, Living People Who Breathe and Feel and Suffer and Love

**Figure:**
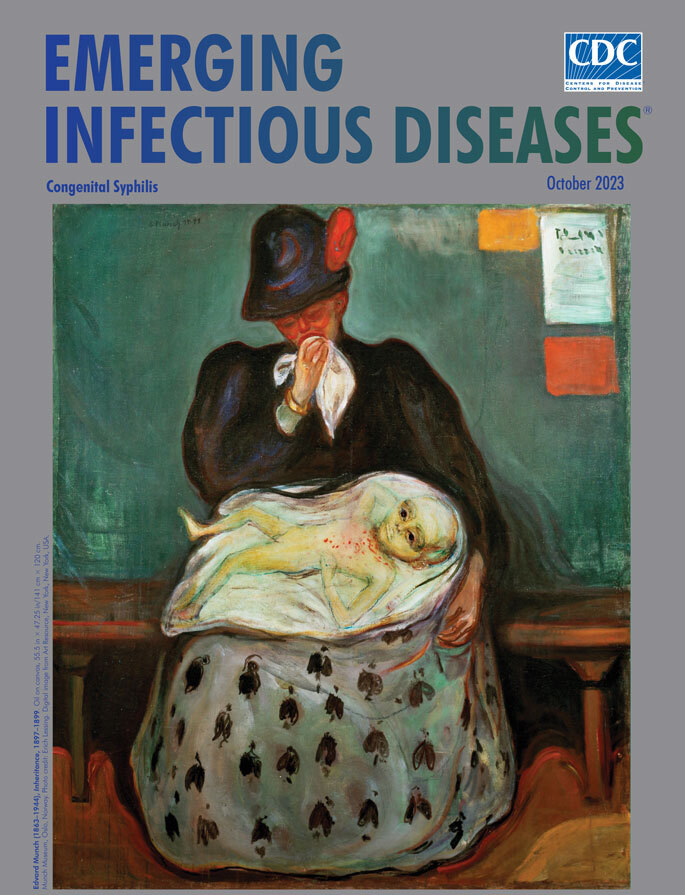
**Edvard Munch (1863–1944), *Inheritance*, 1897–1899.** Oil on canvas, 55.5 in × 47.25 in/141 cm × 120 cm. Munch Museum, Oslo, Norway. Photo credit: Erich Lessing. Digital image from Art Resource, New York, New York, USA.

Since its initial appearance in Europe during the 1400s, syphilis has been a scourge across all levels of society, and it remains one of the most common sexually transmitted infections around the world. The Centers for Disease Control and Prevention (CDC) reports that after the rate of primary and secondary syphilis in the United States dipped to historic lows in 2000 and 2001, the rate has increased almost every year since then; the annual increase for 2020–2021 was 29%. The World Health Organization estimated that, globally in 2020, approximately 7.1 million adults, 15–49 years of age, acquired syphilis. 

Rates of infections with congenital syphilis, which is syphilis transmitted to a fetus either before or during birth from an infected mother, are escalating at an even faster pace. The CDC reports that, in 2021, the US national rate of reported congenital syphilis was 78 cases per 100,000 live births, an increase of 31% from 60 cases per 100,000 live births in 2020. The World Health Organization reports: “Congenital syphilis is the second leading cause of preventable stillbirth globally, preceded only by malaria.”

A pair of articles from this issue of Emerging Infectious Diseases provide more data about those soaring rates. MacKenzie, McEvoy, and Ford document the resurgence of sexually transmitted and congenital syphilis across many high-income countries and describe prevention efforts. Staneva, Hobbs, and Dobbs report that, in Mississippi, congenital syphilis rates have exploded in recent years, increasing 1,000% from 2016 to 2021. 

Congenital syphilis can be prevented if a mother is adequately treated for syphilis at least one month before delivery. Early detection is also critical because infants can be treated with penicillin during their first days of life. If untreated, congenital syphilis may cause death for neonates or a range of devastating birth defects and neurologic deficits in infected infants. This month’s cover image, *Inheritance*, by Norwegian artist Edvard Munch, bears witness to some of those effects. 

Munch completed this painting in the late 1890s after visiting Hôpital Saint-Louis, one of three hospitals in Paris that accepted patients with syphilis. There, he encountered a grieving young woman and her infected child. Several years earlier Munch had written, “No longer shall I paint interiors with men reading and women knitting. I will paint living people who breathe and feel and suffer and love.” In *Inheritance*, and several other paintings, he made good on that declaration. The Munch Museum in Oslo, Norway, where this painting is housed, notes that Munch used a similar palette of red, green, black, and white in several paintings, including this one, that dealt with illness, death, and suffering. 

The young mother, herself a victim of syphilis, sitting alone on a narrow wooden bench in a drab waiting room, daubs at her lips with a white handkerchief as her left arm dangles limply. Her flushed face, red from grief and very likely from a syphilitic rash, contrasts with her dark hat and jacket. Art historian Shelley Wood Cordulack notes, “the bright red plume on the mother’s hat accentuates the acuteness of both disease and emotional trauma.” The patterns in her skirt represent falling leaves, a symbol of death, which, as Cordulack writes, Munch also incorporated into a dress his sister is wearing in an 1892 portrait and again in his 1893 painting *Death in the Sickroom*. 

The unswaddled newborn lying on a shimmering gossamer blanket that surrounds him like an ectoplasmic apparition is sprawled across his mother’s lap. Munch takes pains to show how the pale, lethargic infant has been ravaged by congenital syphilis. The child’s torso is covered with red pustules, his thin arms are folded into triangles, he is jaundiced, and his blank eyes peer from a malformed head. 

Physician Antonio Perciaccante and researcher Alessia Coralli wrote, “The work shocked society because Munch had depicted a person with a sexually transmitted disease, a taboo of that time.” They also pointed out, “The portrait’s title is very interesting. It’s a reminder that, in the 1890s, syphilis in neonates was assumed to be an hereditary disease.” How syphilis was transmitted to infants, they add, was a subject of vigorous debate: “the hereditists supported the transmission by paternal sperm, whereas the contagionists stated that the infection was propagated by maternal way.” 

In 1905, approximately 7 years after Munch finished *Inheritance,* a discovery settled that debate. German scientists Fritz Schaudinn and Erich Hoffmann identified the causative organism of syphilis as the bacterium *Treponema pallidum*, and the term “congenital syphilis” entered the medical lexicon. Breakthroughs and refinements in diagnostic methods followed, and in 1943, the year before Munch died, penicillin was found to be an effective treatment for syphilis. 

By the end of the 20th Century, public health aspirations to eliminate syphilis seemed within reach. CDC’s 1999 National Plan to Eliminate Syphilis from the United States expresses that optimism: “As we approach the end of the 20th century, the United States is faced with a unique opportunity to eliminate syphilis within its borders. Syphilis is easy to detect and cure, given adequate access to and utilization of care. Nationally, it is at the lowest rate ever recorded and it is confined to a very limited number of geographic areas.” 

Public health funding to support surveillance, testing, and treatment for sexually transmitted infections, including syphilis, has not been prioritized or sustained. Consequently, somber scenes such as the one Munch immortalized more than 125 years ago still occur in waiting rooms and clinics across the United States and parts of the world.
